# The mTOR effectors 4EBP1 and S6K2 are frequently coexpressed, and associated with a poor prognosis and endocrine resistance in breast cancer: a retrospective study including patients from the randomised Stockholm tamoxifen trials

**DOI:** 10.1186/bcr3557

**Published:** 2013-10-17

**Authors:** Elin Karlsson, Gizeh Pérez-Tenorio, Risul Amin, Josefine Bostner, Lambert Skoog, Tommy Fornander, Dennis C Sgroi, Bo Nordenskjöld, Anna-Lotta Hallbeck, Olle Stål

**Affiliations:** 1 Department of Clinical and Experimental Medicine, Division of Oncology, Linköping University, County Council of Östergötland, Linköping, SE-58185, Sweden; 2 Department of Pathology and Cytology, Karolinska University Hospital, Solna, Stockholm, SE-17176, Sweden; 3 Department of Oncology, Karolinska University Hospital, Stockholm South General Hospital, Stockholm, SE-11883, Sweden; 4 Department of Pathology, Molecular Pathology Research Unit, Massachusetts General Hospital, Boston, MA, 02129, USA

## Abstract

**Introduction:**

mTOR and its downstream effectors the 4E-binding protein 1 (4EBP1) and the p70 ribosomal S6 kinases (S6K1 and S6K2) are frequently upregulated in breast cancer, and assumed to be driving forces in tumourigenesis, in close connection with oestrogen receptor (ER) networks. Here, we investigated these factors as clinical markers in five different cohorts of breast cancer patients.

**Methods:**

The prognostic significance of 4EBP1, S6K1 and S6K2 mRNA expression was assessed with real-time PCR in 93 tumours from the treatment randomised Stockholm trials, encompassing postmenopausal patients enrolled between 1976 and 1990. Three publicly available breast cancer cohorts were used to confirm the results. Furthermore, the predictive values of 4EBP1 and p4EBP1_S65 protein expression for both prognosis and endocrine treatment benefit were assessed by immunohistochemical analysis of 912 node-negative breast cancers from the Stockholm trials.

**Results:**

S6K2 and 4EBP1 mRNA expression levels showed significant correlation and were associated with a poor outcome in all cohorts investigated. 4EBP1 protein was confirmed as an independent prognostic factor, especially in progesterone receptor (PgR)-expressing cancers. 4EBP1 protein expression was also associated with a poor response to endocrine treatment in the ER/PgR positive group. Cross-talk to genomic as well as non-genomic ER/PgR signalling may be involved and the results further support a combination of ER and mTOR signalling targeted therapies.

**Conclusion:**

This study suggests S6K2 and 4EBP1 as important factors for breast tumourigenesis, interplaying with hormone receptor signalling. We propose S6K2 and 4EBP1 as new potential clinical markers for prognosis and endocrine therapy response in breast cancer.

## Introduction

The outcome of breast cancer patients has been considerably improved in recent years, as a result of early diagnosis and improved treatment regimens; however, breast cancer remains a leading cause of malignancy-associated death among women worldwide. Traditionally, breast cancers have been classified into prognostically meaningful groups based on clinical features and histopathological findings, but it is increasingly evident that cellular and molecular characteristics are of significant importance.

Oestrogen receptor alpha (ER), expressed in 70 to 80% of breast cancers, is a standard biomarker for prediction of response to endocrine treatment. However, significant proportions of ER-positive tumours are resistant to endocrine therapy, either *de novo* or acquired, and more specific biomarkers as well as new therapeutic targets for endocrine-resistant tumours are needed. Suggested mechanisms of endocrine resistance include loss of ER expression or expression of truncated ER isoforms, posttranslational modification of the ER, deregulation of cofactors, or overstimulation of tyrosine kinase receptor growth signalling pathways [1].

The serine/threonine kinase mammalian/mechanistic target of rapamycin (mTOR) is assumed to be a critical effector for several cellular functions deregulated in cancer [2]. mTOR exists in two cellular complexes, referred to as mTORC1 and mTORC2. In response to growth factors, hormones, nutrients, hypoxia and energy/ATP, mTORC1 regulates cell growth, proliferation and metabolism through translational control of essential proteins. The most well-known substrates of mTORC1 are the 4E-binding protein 1 (4EBP1) and the p70 ribosomal S6 kinases 1 and 2 (S6K1 and S6K2), which are involved in regulation of the translational machinery [2]. Two major regulators of mTORC1 function, the rat sarcoma oncogene/mitogen-activated protein kinase and phosphatidylinositol-3-kinase (PI3K)/AKT signalling pathways are constitutively activated in many cancers; however, the mechanisms behind mTORC2 activation are less known. mTORC2 has been shown to be phosphorylated and activated in response to growth factors, but the intracellular pathways remain to be unravelled. The complex has been implicated in cytoskeletal dynamics, through activation of Rho GTPases and PKCα, but also in regulation of AKT through direct phoshorylation of Ser473, thereby promoting its activation [2].

The most frequently altered intracellular growth signalling pathway in breast cancer is PI3K/AKT/mTOR, which is suggested as a key driver of proliferation and survival, particularly in ER-positive tumours. PI3K/AKT/mTOR and ER are implicated in a bidirectional cross-talk, in which intracellular signalling pathways stimulate genomic ER signalling through phosphorylation and activation of the receptor and its cofactors. In addition, oestrogen stimulation of breast cancer cells immediately upregulates intracellular kinase signalling, suggesting nongenomic signalling through cytoplasmic or membrane bound ER to be involved in activation of PI3K/AKT/mTOR signalling [3]. Targeting mTOR has emerged as a new promising treatment strategy for several malignancies and recent data indicate that combining endocrine therapy in breast cancer with mTOR inhibitors is effective [4,5].

Studies have indicated the importance of alterations in factors downstream of mTOR for the development of malignancy. S6K1 as well as S6K2 have been shown to be upregulated in breast cancer [6]. The genes *RPS6KB1* (*S6K1*) and *RPS6KB2* (*S6K2*) are situated in the chromosomal regions 17q21-23 and 11q13, which are commonly amplified in several malignancies. *S6K1* amplification and S6K1 protein overexpression have previously been associated with a worse outcome in breast cancer [7-9]. We have also recently shown that *S6K2* is amplified and overexpressed in breast tumours, and the results indicated that *S6K1* and *S6K2* amplification may have prognostic significance independent of the neighbouring oncogenes *ERBB2* and *CCND1*[8].

Phosphorylation of 4EBP1 by mTORC1 promotes dissociation of 4EBP1 from EIF4E, enabling EIF4E to induce protein translation. Consequently, phosphorylated 4EBP1 (p4EBP1) has been generally accepted as a marker of activated mTOR signalling and high levels in tumours have been associated with a worse outcome in several malignancies, whereas nonphosphorylated 4EBP1 has been considered a tumour suppressor [10]. However, the gene encoding 4EBP1 is located at the chromosomal region 8p12, which is commonly amplified in breast cancer, and in a recent study gene amplification and high mRNA levels of 4EBP1 were shown to indicate a poor prognosis [11]. This suggests that 4EBP1 may have an active role in carcinogenesis. Accordingly, 4EBP1 has also been shown to bind and stabilise mTORC1, promoting activation of the signalling pathway [12].

The mTORC1/S6K/4EBP1 pathway is a major regulator of protein synthesis by phosphorylating several factors in the translational initiation complex, and is thus considered as mainly acting in the cytoplasm [13]. However, recent studies have shown that mTOR as well as the S6 kinases and 4EBP1 can shuttle between the cytoplasm and the nucleus, and are indicated to be involved in regulation of transcription [14-17].

The present aim was to further investigate the significance of 4EBP1 together with S6K1 and S6K2 in breast cancer, in a study encompassing five different cohorts of patients. We showed that S6K2 and 4EBP1 have a correlated mRNA expression, and that high levels of S6K2 and/or 4EBP1 were associated with a poor prognosis, independently of other classical prognostic markers. Furthermore, high cytoplasmic levels of 4EBP1 protein predicted a poor prognosis, whereas 4EBP1 expression, regardless of cellular location, was associated with a decreased benefit from endocrine treatment, suggesting a new role for 4EBP1 in hormone receptor signalling. This study establishes the mTOR effectors 4EBP1 and S6K2, as new potential clinical markers in breast cancer diagnostics and treatment prediction.

## Methods

The study encompasses two cohorts from the randomised adjuvant Stockholm tamoxifen trials, referred to as Stockholm 2 and Stockholm 3. In addition, three publically available datasets were used to confirm the results. The design of the present study and the results presentation are in line with the Reporting Recommendations for Tumour Marker Prognostic Studies guidelines [18].

### Patients in the randomised Stockholm tamoxifen trials

The Stockholm 2 and Stockholm 3 cohorts consist of postmenopausal breast cancer patients enrolled in randomised adjuvant studies between November 1976 and April 1990. Study designs and long-term follow-up data were previously reported in detail [19,20]. Briefly, patients in the Stockholm 2 cohort had positive lymph nodes and/or a tumour diameter exceeding 30 mm, whereas the Stockholm 3 cohort consisted of breast cancer patients with a tumour diameter ≤30 mm and no lymph node involvement. All patients were randomised to receive tamoxifen for 2 years or no endocrine treatment. Patients in the Stockholm 2 cohort were further randomised to postoperative radiotherapy or cyclophosphamide–methotrexate–5-fluorouracil-based chemotherapy. Most of the patients in the tamoxifen arm, if disease free after 2 years, were then randomised to receive tamoxifen for 3 years more or no further adjuvant treatment. Patient flow through the study is presented in Additional file 1: Figure S1 and in Additional file 2. Clinicopathological data can be found in Additional file 3. For the present study, 93 and 912 tumour samples were available from the Stockholm 2 and Stockholm 3 cohorts, respectively. Tumour characteristics and treatments were comparable with the original cohort.

Ethical approval for the Stockholm 2 and Stockholm 3 cohorts was from Karolinska Institute Ethics Council (Dnr KI 97–451 with amendment 030201). Retrospective studies of biomarkers were approved by the local ethics board at the Karolinska Institute, Stockholm, Sweden. Further need for patient consent was waived by the ethical review board.

### RNA extraction and real-time polymerase chain reaction (Stockholm 2)

Fresh-frozen tumour tissue, estimated to contain >50% cancer cells, was homogenised with a microdismembrator (B. Braun Melsungen AG, Germany) or a tissue lyser (Qiagen Hilden, Germany) and total RNA was isolated with the mirVana™ miRNA isolation kit (Ambion Life Technology, Grand Island, NY, USA), according to instructions provided by the manufacturers. Purified RNA was dissolved in nuclease-free water with addition of RNAsin Ribonuclease inhibitor (Promega Madison, WI, USA) and was stored at -70°C. RNA integrity numbers and concentrations were assessed with an Agilent 2100 Bioanalyser (Agilent Biosystems Santa Clara, CA, USA). Only samples with RNA integrity numbers ≥5 were included in the analysis.

Reverse transcription was performed using the high-capacity cDNA reverse transcription kit (Applied Biosystems Foster City, CA, USA) with 200 ng total RNA in reactions of 20 μl according to the manufacturer’s instructions. mRNA expression of S6K1, S6K2 and 4EBP1 was quantified with fast real-time polymerase chain reaction (PCR) using an ABI Prism 7900ht (Applied Biosystems). TaqMan assays (Applied Biosystems) for S6K1 (Hs00177357_m1), S6K2 (Hs00177689_m1), 4EBP1 (Hs0060705_m1) and the endogenous controls β-actin (ACTB; part number 4310881E) and peptidylprolyl isomerase A (cyclophilin A) (PPIA; part number 4333763 F) were handled according to the manufacturer’s instructions. Quantitative PCR was performed in duplicate with 10 μl reaction volume in 1× TaqMan fast universal master mix (Applied Biosystems) using the following thermal conditions: 95°C for 20 seconds; 40 cycles of 95°C for 1 second, and 60°C for 20 seconds. To confirm specificity, reactions without reverse transcriptase as well as no template controls were included on each plate. The mean value was taken from the duplicates and relative expression was calculated with the ΔΔCt method, using SKBR3 cDNA as the calibrator. For the two endogenous controls, an average value for each sample was used. For correlation analyses, expression levels of the genes were divided into four groups based on the quartiles. In the survival analyses, the upper quartile was considered as high expression and the remaining levels as low expression, if nothing else is specified.

### Tissue microarray preparation and immunohistochemical analysis (Stockholm 3)

The protein expressions of total 4EBP1 and 4EBP1 phosphorylated at Serine 65 (hereafter referred to as 4EBP1 and p4EBP1, respectively) were evaluated in the Stockholm 3 cohort by immunohistochemical (IHC) staining of tissue microarrays. Core needle biopsies from paraffin-embedded tissues were reembedded in new paraffin blocks and the blocks were cut into 4 μm sections and mounted on frost-coated slides. The slides were deparaffinised in xylene and rehydrated in decreasing concentrations of ethanol, and antigen retrieval was performed in citrate buffer (pH 6.0) in a pressure cooker with the default program 125°C for 30 seconds followed by 90°C for 10 seconds at a pressure of 23 to 25 psi. Endogenous peroxidases were blocked with 3% H_2_O_2_ in MeOH for 5 minutes, and protein block X0909 (Dako Glostrup, Denmark) was applied for 10 minutes to reduce unspecific binding. The slides were incubated with primary antibodies for 4EBP1 (53H11/#9644, diluted 1:100; Cell Signaling Danvers, MA, USA) or p4EBP1_S65 (174A9/#9456, diluted 1:100; Cell Signaling) overnight at 4°C. Secondary antibody (EnVision™; Dako) was applied for 30 minutes at room temperature. For visualisation, the slides were incubated in 3,3′-diaminobenzidine hydrochloride/H_2_O_2_ for 8 minutes at room temperature and in darkness, and counterstained with haematoxylin for 1 minute at room temperature and in darkness. Representative images of the stainings were photographed at 40× magnification using an Olympus SC20 digital camera (Olympus Järfälla, Stockholm, Sweden) connected to a Leica LB30T microscope (Leica Microsystems Kista, Stockholm, Sweden) (Additional file 1: Figures S2 and S3). Phospho-specificity for p4EBP1_S65 was evaluated with lambda phosphatase (New England Biolabs Ipswitch, UK) according to manufacturer’s instructions (Additional file 1: Figure S3). Protein specificity of the 4EBP1 antibodies was validated with western blot, by us and others [16,21] (Additional file 1: Figures S2 and S3). Cytoplasmic and nuclear intensity of the stainings was evaluated by two independent observers, according to the levels depicted in Additional file 4. In the survival analyses, a high 4EBP1 expression was defined as strong cytoplasmic or nuclear staining, whichever indicated. The variable 4EBP1cytoplasm ≥ nucleus was defined as a cytoplasmic staining stronger than or equal to the nuclear staining detected.

### Evaluation of other clinicopathological variables

ER expression was determined at the time of diagnosis, before 1988 using isoelectric focusing and after that with quantitative enzyme immunoassay [19,20]. In the Stockholm 3 cohort, where tissue microarrays were available, the ER and progesterone receptor (PgR) status was further determined retrospectively by IHC using the Ventana automated slide stainer (Ventana Medical Systems Tucson, Arizona) with monoclonal Ventana CONFIRM mouse primary ER and PgR antibodies [22]. The cutoff level for ER and PgR positivity was >10% stained nuclei or, when IHC data were not available, 0.05 fmol/μg DNA. Isoelectric focusing/enzyme immunoassay and IHC data have been shown to be comparable [23]. In the Stockholm 2 cohort, human epidermal growth factor receptor 2 (HER2) protein was quantified retrospectively by flow cytometry [24] and HER2 amplification was determined with quantitative real-time PCR [25]. HER2 protein expression in the Stockholm 3 cohort was evaluated with IHC as described elsewhere [26], whereas tumour grade was evaluated retrospectively according to the Nottingham system [22]. In the Stockholm 2 cohort, S-phase fraction was previously determined by flow cytometry [27]. Extraction of DNA from fresh-frozen tissue and analysis of the *S6K1* and *S6K2* gene copy number were described elsewhere [8,11]. Analyses of mutations in *PIK3CA* as well as protein expression of pAKT_S473 in the Stockholm 2 cohort were reported earlier [28,29]. In the Stockholm 3 cohort, the S6K2, pAKT_S473 and pmTOR_S2448 IHC stainings have also been described previously [8,30].

### Public datasets (van de Vijver, Uppsala, Karolinska)

Public available datasets encompassing preprocessed mRNA expression data were downloaded for three cohorts, further referred to as the van de Vijver cohort (*n* = 295) [31], the Uppsala cohort (*n* = 236) [NCBI/GEO:GSE3494] and the Karolinska Institute cohort (*n* = 159) [NCBI/GEO:GSE1456]. Patient flow is overviewed in Additional file 2. The patient characteristics are briefly described in Additional file 3 and were previously presented in detail, as was the data processing method [9,32,33].

### Statistical analysis

Associations between different variables were assessed by Spearman’s rank-order correlation. The Kaplan–Meier product limit method was used to estimate the cumulative probabilities of distant recurrence-free survival or breast cancer-specific survival, and differences between the curves were evaluated with the log-rank test or Gehan’s test for multiple groups. For univariate and multivariate analysis of event rates, as well as interaction analysis, Cox proportional hazard regression was used.

In the interaction test, the Cox model included the variables 'tamoxifen treatment’ and '4EBP1 expression’ and the interaction variable ’tamoxifen treatment × 4EBP1 expression’. All statistical analyses were performed with Statistica 9.0 (Statsoft, Inc. Uppsala, Sweden) and *P* <0.05 was considered statistically significant, with exception of the correlation analyses where *P* <0.01 was applied to compensate for multiple testing.

## Results

### Gene amplifications of S6K1 and S6K2 are associated with high levels of corresponding mRNA

4EBP1, S6K1 and S6K2 mRNA levels were quantified in 93 tumours from the Stockholm 2 cohort. *S6K1* and *S6K2* gene amplification was previously determined with real-time PCR in 206 and 207 breast tumour samples, respectively [8]. There was a significant correlation between gene copy number and mRNA levels for both genes (*S6K1*: Spearman *R* = 0.30, *P* = 0.007; *S6K2*: Spearman *R* = 0.43, *P* = 0.0001). An increased gene copy number was almost always accompanied by high mRNA levels, but high mRNA levels could be detected in additional samples, independent of gene copy status (Additional file 5).

### 4EBP1 mRNA is frequently coexpressed with S6K2, but not with S6K1

**Figure 1:**
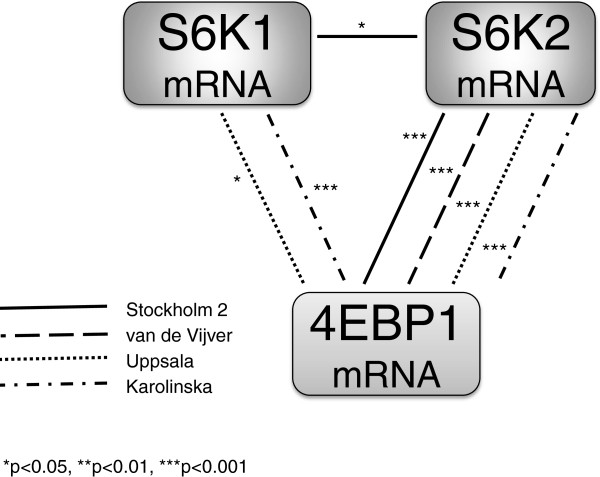
**Associations between p70 ribosomal S6 kinase 1 and 2 and 4E-binding protein 1 mRNA expression.** Spearman’s rank**-**order correlation evaluating associations between p70 ribosomal S6 kinase 1 (S6K1), p70 ribosomal S6 kinase 2 (S6K2) and 4E-binding protein 1 (4EBP1) mRNA expression (continuous values) in four breast cancer cohorts.

In a previous study encompassing 29 of the Stockholm 2 patients, S6K2 and 4EBP1 were found to be coamplified and expression levels for the corresponding mRNAs were correlated [11]. In line with this finding, when considering all 93 patients in the present study, S6K2 and 4EBP1 mRNA levels were significantly correlated (Spearman *R* = 0.32, *P* = 0.0018). There was no correlation between S6K1 and 4EBP1 mRNA levels (Spearman *R* = -0.0017, *P* = 0.99; Figure 1). S6K1 mRNA was positively correlated with ER status (Spearman *R* = 0.39, *P* = 0.00009; Additional file 5). There was also an inverse association between high S6K1 mRNA levels and HER2 amplification/protein levels as well as high S-phase fraction (Additional file 5). A correlation between S6K2 and 4EBP1 mRNA expression could be confirmed in the three public cohorts, whereas S6K1 and 4EBP1 mRNA levels were associated with high significance in the Karolinska cohort only (Figure 1). The association between S6K1 and ER status in Stockholm 2 could not be detected in the other cohorts (data not shown).

### High mRNA levels of S6K2 and 4EBP1 are associated with an adverse outcome in four breast cancer cohorts

**Figure 2:**
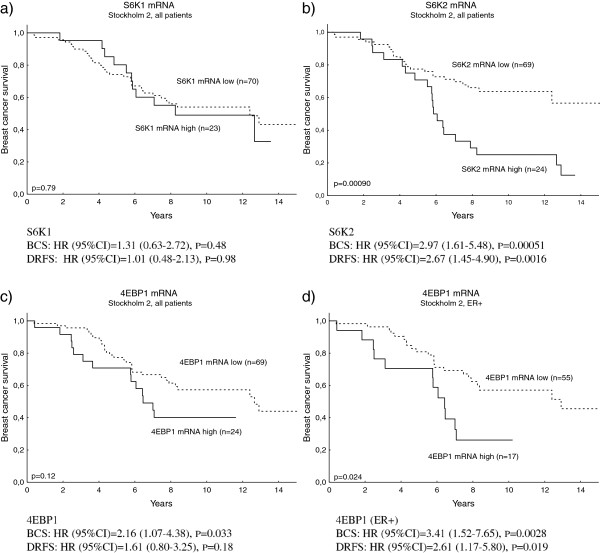
**Breast cancer survival and distant recurrence-free survival in the Stockholm 2 patient cohort in relation to S6K1, S6K2 and 4EBP1 mRNA levels.** Kaplan–Meier curves and multivariate Cox regression of breast cancer survival (BCS) and distant recurrence-free survival (DRFS) in the Stockholm 2 patient cohort, in relation to **(a)** p70 ribosomal S6 kinase 1 (S6K1) mRNA, **(b)** p70 ribosomal S6 kinase 2 (S6K2) mRNA, **(c)** 4E-binding protein 1 (4EBP1) mRNA, and **(d)** 4EBP1 mRNA in the oestrogen receptor alpha (ER**)**-positive subgroup. The Cox analysis included the following variables: adjuvant chemotherapy treatment, endocrine treatment, lymph node status, tumour size, and ER status. CI, confidence interval; HR, hazard ratio.

**Figure 3:**
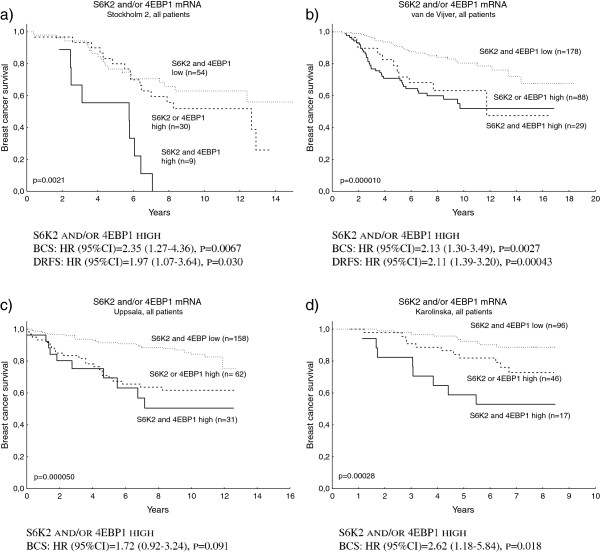
**Breast cancer survival and distant recurrence-free survival in relation to high S6K2 and/or 4EBP1 mRNA in four cohorts.** Kaplan–Meier curves and multivariate Cox regression of breast cancer survival (BCS) and, when available, of distant recurrence-free survival (DRFS) in relation to high p70 ribosomal S6 kinase 2 (S6K2) and/or 4E-binding protein 1 (4EBP1) mRNA in four cohorts: **(a)** Stockholm 2, **(b)** van de Vijver, **(c)** Uppsala, and **(d)** Karolinska. The variable S6K2 and/or 4EBP1 was defined in two groups as highest quartile mRNA expression of: neither S6K2 nor 4EBP1 (=0), or of S6K2 and/or 4EBP1 (=1). The Cox analysis also included the following variables: adjuvant chemotherapy treatment, endocrine treatment, lymph node status, tumour size (with exception of van de Vijver), and oestrogen receptor alpha status. CI, confidence interval; HR, hazard ratio.

*S6K1*, *S6K2* and *4EBP1* gene amplification have earlier been connected to a worse prognosis in breast cancer. At the mRNA level, S6K2 and 4EBP1 remained independent prognostic factors in the Stockholm 2 cohort, whereas this could not be seen for S6K1 (Figure 2a,b,c). For 4EBP1, the prognostic value was especially pronounced in the ER-positive subgroup (Figure 2d). A combination variable of high S6K2 and/or 4EBP1 mRNA was a significant independent prognostic factor, and the worst outcome could be seen in the group with the highest levels of both S6K2 and 4EBP1 (Figure 3a). The prognostic value of S6K1, S6K2 and 4EBP1 mRNA was further analysed in the three public cohorts (Additional file 1: Figures S4, S5 and S6). 4EBP1 remained an independent prognostic factor in the van de Vijver and Karolinska cohorts. S6K2 was also significantly associated with clinical outcome in the Karolinska cohort and, when divided into two groups based on the median, this was also true in the van de Vijver cohort. In the Uppsala cohort, S6K2 and 4EBP1 remained prognostic factors in the univariate analysis, whereas the multivariate analyses did not reach significance. S6K1 was significantly associated with a worse outcome in the van de Vijver cohort only. The combined variable S6K2 and/or 4EBP1 mRNA was confirmed as a significant prognostic factor, related to poor outcome, in the van de Vijver and Karolinska cohorts, and a borderline significance was seen in the Uppsala cohort (Figure 3b,c,d).

There was a significant correlation between high S6K2 and/or 4EBP1 to grade in the Uppsala and Karolinska cohorts as well as to the proliferation marker cyclin A_2_ in the van de Vijver cohort. In the Stockholm 2 cohort, the correlation between S6K2 and/or 4EBP1 and high S-phase fraction reached borderline significance. High S6K2 and/or 4EBP1 was primarily seen in ER/PgR-negative tumours in the van de Vijver and Uppsala cohorts and the same tendency could be seen in the Karolinska cohort. High S6K2 and/or 4EBP1 was also significantly associated with large tumour size in the Uppsala material (Table 1).

**Table 1:** Association between high S6K2 and/or 4EBP1 mRNA and clinicopathological parameters in the four cohorts

**S6K2 and/or 4EBP1 mRNA (Spearman *R*, *P *** **value)**				
	**Stockholm 2**	**Van de Vijver**	**Uppsala**	**Karolinska**
	***(n = 93)***	***(n = 295)***	***(n = 251)***	***(n = 159)***
Lymph node status	*R* = -0.11	*R* = 0.07	*R* = 0.31	*R* = -0.07
	*P* = 0.25	*P* = 0.21	*P* = 0.000001	*P* = 0.36
Tumour size	*R* = -0.12	N/A	*R* = 0.32	*R* = 0.16
	*P* = 0.23		*P* < 0.0000001	*P* = 0.040
Grade/proliferation^a^	*R* = 0.23	*R* = 0.42	*R* = 0.45	*R* = 0.41
	*P* = 0.034	*P* < 0.0000001	*P* <0.0000001	*P* = <0.0000001
ER status	*R* = -0.01	*R* = -0.31	*R* = -0.26	*R* = -0.11
	*P* = 0.91	*P* <0.0000001	*P* = 0.000046	*P* = 0.15
PgR status (protein)	N/A	N/A	*R* = -0.27	N/A
			*P* = 0.000010	
PgR mRNA	N/A	*R* = -0.29,	*R* = -0.30	*R* = -0.16
		*P* <0.0000001	*P* = 0.000002	*P* = 0.048

Spearman’s rank-order correlation evaluating the association between high S6K2 and/or 4EBP1 mRNA and clinicopathological parameters in the four cohorts. The variable S6K2 and/or 4EBP1 was defined in three groups as highest quartile mRNA expression of: neither S6K2 nor 4EBP1 (=0), either S6K2 or 4EBP1 (=1), or S6K2 and 4EBP1 (=2). 4EBP1, 4E-binding protein 1; ER, oestrogen receptor alpha; PgR, progesterone receptor; S6K2, p70 ribosomal S6 kinase 2.

^a^Stockholm 2, S-phase fraction; van de Vijver, cyclin A_2_ mRNA expression; Uppsala and Karolinska, Elston grade. N/A, not available.

### Clinicopathological characteristics of 4EBP1-expressing tumours are dependent on the cellular localisation of the protein

Protein expression of 4EBP1 and p4EBP1 could be analysed in 739 and 768 tumours, respectively, in the Stockholm 3 cohort. 4EBP1 and p4EBP1 were detected in both the nucleus and the cytoplasm of the tumour cells (Additional file 4). Correlations between 4EBP1 and p4EBP1 protein expression and clinicopathological factors are described in Additional file 6. Strong cytoplasmic 4EBP1 and p4EBP1 expression was associated with high-grade and HER2-positive tumours and also with large tumour size. Nuclear p4EBP1 was associated with small, low-grade tumours. Nuclear and cytoplasmic p4EBP1 were significantly correlated with pAKT expression in the respective compartments. There was no significant correlation between pmTOR and p4EBP1 or 4EBP1. Both p4EBP1 and cytoplasmic 4EBP1 were significantly associated with S6K2 protein expression (Additional file 6).

### p4EBP1 and 4EBP1 protein expression are independent prognostic factors in breast cancer

**Figure 4:**
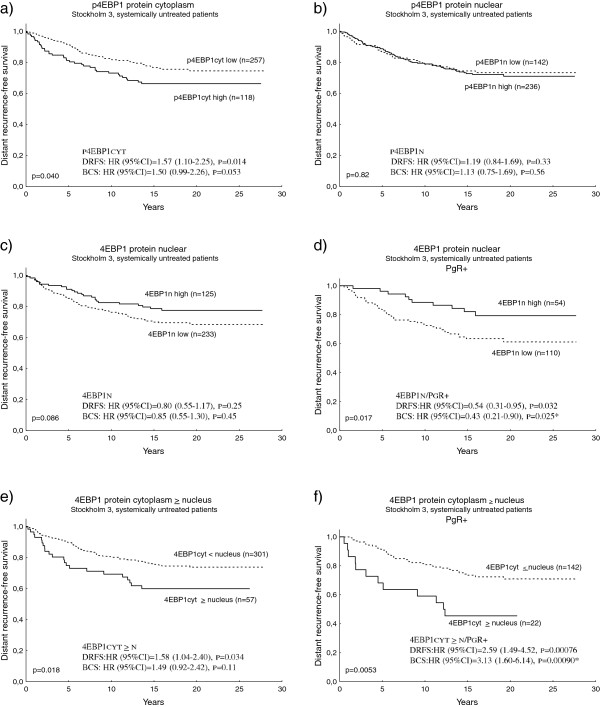
**Survival among systemically untreated patients in Stockholm 3 in relation to p4EBP1_S65 and 4EBP1 protein.** Kaplan–Meier curves and multivariate Cox regression of distant recurrence-free survival (DRFS) and breast cancer survival (BCS) among systemically untreated patients in the Stockholm 3 cohort in relation to **(a)** p4EBP1_S65 cytoplasmic protein, **(b)** p4EBP1_S65 nuclear protein, **(c)** 4E-binding protein 1 (4EBP1) nuclear protein, **(d)** 4EBP1 nuclear protein in the progesterone receptor (PgR)-positive subgroup, **(e)** 4EBP1 protein cytoplasm ≥ nucleus, and **(f)** 4EBP1 protein cytoplasm ≥ nucleus in the PgR-positive subgroup. The Cox analysis included the following variables: adjuvant tamoxifen treatment, tumour size, Nottingham histological grade, oestrogen receptor alpha, and human epidermal growth factor receptor 2 (HER2) status. *For the PgR-positive/BCS multivariate analyses, HER2 was divided into four groups due to few events. CI, confidence interval; HR, hazard ratio.

High tumour levels of p4EBP1 have earlier been associated with poor outcome in breast cancer and other malignancies. For systemically untreated patients, in the present study, strong cytoplasmic p4EBP1 staining remained an independent prognostic factor, predicting decreased distant recurrence-free survival and poor breast cancer survival (Figure 4a). In contrast, nuclear p4EBP1 did not correlate with prognosis (Figure 4b), while strong nuclear 4EBP1 staining indicated good prognosis (Figure 4c), and this was especially evident in the PgR-positive subgroup (Figure 4d). No prognostic significance could be seen for cytoplasmic 4EBP1 (data not shown), but the variable 4EBP1cytoplasm ≥ nucleus was an independent prognostic factor, predicting increased risk of distant recurrence and breast cancer death (Figure 4e), especially among patients with PgR-expressing tumours (Figure 4f).

### High cytoplasmic protein levels of 4EBP1 predict a decreased benefit from endocrine treatment

**Figure 5:**
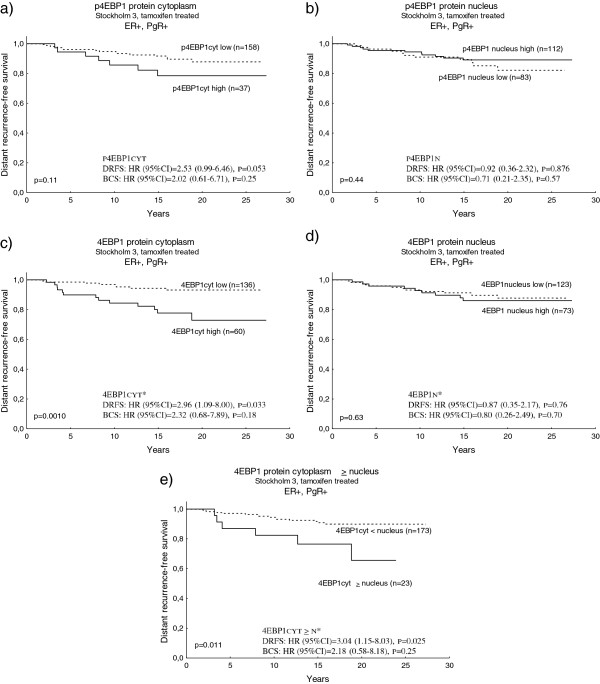
**Distant recurrence-free survival among patients in Stockholm 3 with ER-positive/PgR-positive tumours treated with tamoxifen in relation to p4EBP1_S65 and 4EBP1 protein expression.** Kaplan–Meier curves and multivariate Cox regression of distant recurrence-free survival (DRFS) among patients in the Stockholm 3 cohort with ER-positive/PgR-positive tumours, treated with tamoxifen, in relation to **(a)** p4EBP1_S65 cytoplasmic protein, **(b)** p4EBP1_S65 nuclear protein, **(c)** 4E-binding protein 1 (4EBP1) cytoplasmic protein, **(d)** 4EBP1 nuclear protein, and **(e)** 4EBP1protein cytoplasm ≥ nucleus. The Cox analysis included the following variables: tumour size, Nottingham histological grade, and human epidermal growth factor receptor 2 (HER2) status. *For the 4EBP1 analysis, HER2 was divided into four groups due to few events. BCS, breast cancer-specific survival; CI, confidence interval; ER, oestrogen receptor alpha; HR, hazard ratio; PgR, progesterone receptor.

**Figure 6:**
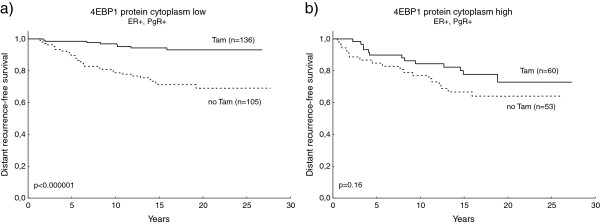
**Distant recurrence-free survival for ER-positive/PgR-positive breast cancer patients in Stockholm 3 treated or not with tamoxifen in relation to 4EBP1 cytoplasmic protein.** Distant recurrence-free survival (DRFS) for breast cancer patients in the Stockholm 3 cohort treated with tamoxifen (Tam) versus no tamoxifen (no Tam) in the ER-positive/PgR-positive subgroup in relation to **(a)** low and **(b)** high cytoplasmic 4E-binding protein 1 (4EBP1) protein levels. ER, oestrogen receptor alpha; PgR, progesterone receptor.

Upregulation of the AKT/mTOR pathway has been implicated as one mechanism behind endocrine resistance. In the Stockholm 3 cohort, the outcome among patients with ER-positive/PgR-positive tumours treated with tamoxifen was evaluated in relation to 4EBP1 protein expression in different compartments (Figure 5). This analysis confirmed cytoplasmic 4EBP1 to be predictive of poor clinical outcome in the tamoxifen-treated ER-positive /PgR-positive group (Figure 5c), as well as the variable 4EBP1 cytoplasm ≥ nucleus (Figure 5e). In addition, cytoplasmic p4EBP1 (Figure 5a) was shown borderline significant in relation to a poor prognosis in this patient group . Nuclear p4EBP1 or nuclear 4EBP1 was not related to outcome after tamoxifen treatment (Figure 5b,d). In a subsequent analysis, the benefit from tamoxifen was compared between patients with ER-positive/PgR-positive tumours expressing low or high cytoplasmic levels of p4EBP1 or 4EBP1. Tamoxifen treatment was associated with a strongly reduced risk of distant recurrence in the group of patients with ER-positive/PgR-positive tumour and low cytoplasmic 4EBP1 (distant recurrence-free survival: hazard ratio (95% confidence interval) = 0.19 (0.09 to 0.42), *P* = 0.00003; Figure 6a), whereas no significant benefit from tamoxifen could be seen in the 4EBP1 high cytoplasmic group (distant recurrence-free survival: hazard ratio (95% confidence interval) = 0.60 (0.30 to 1.23), *P* = 0.17; Figure 6b). The difference in treatment benefit between the groups with low and high cytoplasmic 4EBP1 was significant (test for interaction, *P* = 0.034). The interaction test concerning cytoplasmic p4EBP1 did not reach significance (data not shown).

## Discussion

The role of mTOR signalling in cancer development, progression and as a potential treatment target is increasingly evident. In this study, we highlight the clinical importance of factors downstream of mTOR, and show that mRNA expression of S6K2 and 4EBP1 are correlated and significantly related to poor outcome in four independent breast cancer cohorts. This is the first study showing high 4EBP1 mRNA, independent of phosphorylation status, and cytoplasmic protein levels to be associated with poor prognosis in breast cancer. Furthermore, high 4EBP1 protein levels predicted less benefit from the endocrine treatment tamoxifen, indicating interactions with hormone receptor signalling. This suggests that the mTOR effectors S6K2 and 4EBP1 may be used as prognostic indicators and for treatment prediction.

The S6 kinases are frequently upregulated in breast cancer, and associated with a poor outcome [6,34]. In the present study, we could show a correlation between gene amplification and increased mRNA levels for *S6K1*, *S6K2* as well as seen previously for *4EBP1*[11]. Tumours with amplification of these genes had high levels of the corresponding mRNA; however, high mRNA expression was also in some cases seen in tumours with normal gene copy numbers. Recently, S6K1 was described as a transcriptional target of the ER [35]. Here, there is a correlation between ER and S6K1 mRNA levels in the Stockholm 2 cohort, suggesting that ER expression could be one mechanism behind S6K1 upregulation in breast tumours. However, *S6K1* gene amplification in Stockholm 2 was in a previous study correlated with HER2 positivity rather than ER expression [8], probably as a consequence of the localisation of the *S6K1* gene in proximity of the *ERBB2* gene at 17q. It is evident that, although amplification and expression of these genes are tightly accompanied, these events are not identical. Gene amplification probably reflects the contribution of several genes in the amplicons, and the feature of expression is highly dependent on the cellular localisation of the proteins.

The previously implicated associations between S6K2 and 4EBP1 [11] were further confirmed in this study, and could be seen in several independent and clinically different patient materials. High S6K2 and/or 4EBP1 mRNA was associated with poor outcome in all investigated cohorts, which may reflect a possible synergy between S6K2 and 4EBP1 in promoting tumourigenesis. p4EBP1 has been shown to predict a poor prognosis in several cancer types [36-39] and the protein was recently described as a key funnel factor in carcinogenesis [10]. In general, p4EBP1 has been considered a marker of mTORC1 signalling and activation of the translational machinery. However, there are indications that 4EBP1 could also play a more active role in tumour progression. In this study, cytoplasmic p4EBP1 was confirmed as a prognostic factor; however, 4EBP1 and 4EBP1 mRNA were also associated with high grade and a poor outcome. The gene encoding 4EBP1 is located in the chromosomal region 8p12, which is frequently amplified in breast cancer. Amplification of 8p12 was associated with high 4EBP1 mRNA levels, suggesting 4EBP1 as a potential oncogene, and amplification of the 4EBP1 gene is one possible mechanism behind its overexpression in tumours. Another suggested pathway is through cMyc-dependent transcription, and amplification or induced expression of cMyc has been shown to promote cMyc binding to the 4EBP1 gene and increase its expression. This in turn leads to inhibition of autophagy and rapamycin resistance [40]. Unfortunately, we have not been able to study the possible relation between 4EBP1 mRNA levels and its corresponding protein expression. A recent review on the issue of regulation of protein expression and its relation to mRNA levels conclude that the abundance of mRNA in general highly reflects the ability to detect protein expression in cells [41]. High mRNA levels of 4EBP1 as well as high cytoplasmic protein levels are both related to a high proliferation and a poor prognosis in the different materials investigated. One could therefore speculate that high mRNA levels may reflect increased cytoplasmic protein levels rather than nuclear, perhaps as a result of increased nuclear-cytoplasmic shuttling in proliferating cells, although the mechanisms behind this are unclear.

Interestingly, the prognostic value of 4EBP1 seems to be dependent on the cellular location of the protein. Nuclear expression was related to a better outcome, indicating that 4EBP1 plays divergent roles in different cellular compartments. A previous study estimated that approximately 30% of the 4EBP1 expressed in cells is located in the nucleus, where it has a role in regulating the availability of EIF4E for the cytoplasmic translational machinery, by retaining EIF4E in the nucleus [16]. High nuclear levels of 4EBP1 would thus inhibit translation and subsequent proliferation, which may explain its relation with a good prognosis. The associations between cytoplasmic 4EBP1 as well as high mRNA levels with high grade and poor prognosis indicate a dual role for this protein. 4EBP1 has recently been implicated in a positive feedback loop by binding and stabilising mTORC1, thereby promoting its activation [12]. In the present study, p4EBP1 expression was correlated with pAKT_S473 but not with pmTOR_S2448, a site associated with mTORC1 [42]. Furthermore, recent studies have indicated additional roles of 4EBP1, independent of mTORC1. Rapalogs, mainly targeting mTORC1, have been shown to completely inhibit pS6K but only to partially inhibit p4EBP1 [2]. In bladder cancer, 4EBP1 was shown to be regulated by PI3K but not through mTORC1 [43], and mTOR-independent 4EBP1 phosphorylation has been associated with resistance to mTOR kinase inhibitors [44]. Additional kinases for 4EBP1 regulation remain to be identified. Upstream factors of the PI3K/AKT pathway are likely candidates. Some studies have shown that mTOR kinase inhibitors block p4EBP1 more effectively than rapalogs [2], suggesting mTORC2 as a candidate in 4EBP1 regulation. In our material, there is a significant correlation between cytoplasmic p21-activated kinase 1 (PAK1) and p4EBP1 (data not shown) and the area around S65 in 4EBP1 is in agreement with the consensus sequence reported for PAK1 [45], adding PAK1 to the list of potential candidates. Interestingly, PAK1 was recently described as involved in mTORC2 mediated AKT_S473 phosphorylation, and the kinase may be a part of the complex [46].

Upregulation of the PI3K/AKT/mTOR pathway has been associated with decreased benefit from endocrine therapies in breast cancer, and recent studies support mTOR inhibitors as promising agents for overcoming endocrine resistance [4]. Also, nuclear S6K2 has been associated with response to endocrine therapy, although dependent on PgR status [8]. In our present study, high cytoplasmic but not nuclear expression of 4EBP1 predicted less benefit from tamoxifen, which reached significance for 4EBP1 but not for p4EBP1. 4EBP1 is regulated by phosphorylation at multiple sites, and the role for the different sites is not totally established. The 4EBP1 antibody used in our study is raised towards a sequence surrounding S112, thus at the very C-terminus of 4EBP1, and recognises both unphosphorylated as well as 4EBP1 phosphorylated at different sites. In addition, the 4EBP1 and p4EBP1_S65 stainings are highly correlated, especially for the cytoplasmic pools of the proteins (Additional file 6), indicating that to some extent the same proteins are detected. This may also reflect that an increase in total protein expression is often accompanied with an increased phosphorylation and activation of the proteins. 4EBP1 activation (foremost by mTOR) may therefore be the reason behind its role in endocrine resistance. Interestingly, in a recently published study, both phosphorylated and total 4EBP1 were related to a poor outcome among patients with ER-positive breast cancers, treated with tamoxifen [47], in keeping with our findings. In that study, protein expression was determined by reverse-phase protein arrays, ruling out the possibility to distinguish between cytoplasmic and nuclear expression.

In the present study, the predictive value for 4EBP1 was especially evident in the ER/PgR-expressing subgroup. In addition, the prognostic significance of 4EBP1 was most prominent in combination with PgR expression, suggesting a possible cross-talk between 4EBP1 and nuclear receptors. The role of progesterone signalling in breast cancer remains controversial. In general, circulating progesterone is considered a risk factor for breast cancer development by promoting cellular proliferation. However, in primary breast cancer, PgR expression is associated with differentiated, less aggressive tumours and a favourable prognosis [48]. Upregulation of the insulin-like growth factor/PI3K/AKT/mTOR pathway is one suggested mechanism behind PgR downregulation in breast cancer, despite a functional ER. In agreement, our study showed an inverse association between S6K2/4EBP1 and PgR mRNA levels, in the three available cohorts. Furthermore, the gene encoding PgR is located at the proximal part of the 11q chromosomal arm, which is commonly deleted in 11q13/8p12 amplified tumours [11]. However, 4EBP1 was recently described as a possible target gene for PgR [49], suggesting the presence of a negative feedback loop downregulating PgR after growth factor pathway stimulation. The function of PgR can be regulated by receptor phosphorylation at multiple sites, through growth factor receptor signalling pathways, and a subpopulation of cytoplasmic PgR has also been shown able to activate kinase cascades, including PI3K/AKT [48]. It is tempting to speculate that a coordinated expression of PgR and cytoplasmic growth signalling factors including S6K2/4EBP1 may facilitate the proliferative and oncogenic role of PgR, promoting tumour progression and therapy resistance. In addition, PgR may in the long run be downregulated through PI3K/AKT/mTOR pathway stimulation and subsequent aberrant ER signalling, leading to acquired endocrine resistance among patients with initially ER/PgR-positive breast cancers.

## Conclusions

Inhibitors of mTOR signalling may have a clinical potential in the management of several malignancies, not least as a complement to ER-targeted therapies in breast cancer. However, the complexity of mTOR signalling is far from unravelled. This study evaluates the clinical value of mTOR effectors in breast cancer. We show that 4EBP1 mRNA expression is correlated with S6K2 mRNA and that high S6K2 and/or 4EBP1 is associated with a poor outcome, in four different cohorts of breast cancer. In addition, high cytoplasmic 4EBP1 protein levels predicted a poor prognosis and a decreased benefit from tamoxifen in a large randomised cohort. In summary, suggested pathways of 4EBP1 are illustrated in Additional file 1: Figure S7. Altogether, we propose the mTOR effectors 4EBP1 and S6K2 as new potential clinical markers in breast cancer.

## Abbreviations

AKT: Protein kinase B/v-akt murine thymoma viral oncogene homologue; CCND1: Cyclin D_1_; 4EBP1: 4E-binding protein 1; ER: Oestrogen receptor alpha; ERBB2: v-erb-b2 erythroblastic leukemia viral oncogene homolog 2; HER2: Human epidermal growth factor receptor 2; IHC: Immunohistochemistry; mTOR: Mammalian/mechanistic target of rapamycin; PAK1: p21-activated kinase 1; PCR: Polymerase chain reaction; p4EBP1: Phosphorylated 4EBP1; PgR: Progesterone receptor; PI3K: Phosphatidylinositol-3-kinase; S6K: p70 ribosomal S6 kinase.

## Competing interests

The authors declare that they have no competing interests.

## Authors’ contributions

EK conceived of the study and was involved in the study design, carried out the quantitative PCR analyses, participated in the IHC grading, performed the statistical analyses including handling of the public datasets, participated in interpretation of the results and drafted the manuscript. GP-T conceived of the study and was involved in the design of the study, carried out the IHC stainings and participated in interpretation of the results. RA was involved in the design of the study, carried out the IHC stainings and participated in interpretation of the results. JB and A-LH participated in the design of the study and participated in interpretation of the results. BN, LS and TF were responsible for the clinical trial, the collection of tumour tissue samples and IHC stainings of steroid hormone and HER2 receptors. DCS performed the histological grading of the tumours from the Stockholm 3 cohort. OS conceived of the study, was responsible for the design and coordination of the study, participated in the statistical analyses and the result interpretation. All authors were involved in reading and revising the draft and approved the final manuscript.

## Supplementary Material

Additional file 1: Figure S1 Showing patient flow through the study: the randomised Stockholm tamoxifen trial, Stockholm 2 and Stockholm 3 cohorts. Tam, tamoxifen; RT, radiotherapy; CMF, cyclophosphamide–metotrexate–5-fluorouracil chemotherapy; TMA, tissue microarray; IHC, immunohistochemistry. **Figure S2** showing examples of tumours graded for 4EBP1 nuclear and cytoplasmic staining: negative/weak **(a)**; intermediate **(b)**; and strong staining **(c)**; and validation of 4EBP1 antibody specificity using immunoblot with MCF7 cell lysate **(d)**. **Figure S3** showing examples of tumours graded for p4EBP1_S65 nuclear and cytoplasmic staining: negative/weak **(a)**; intermediate **(b)** and strong staining **(c)**; and validation of p4EBP1_S65 antibody specificity; immunoblot using MCF7 cell lysate **(d)**; p4EBP1 breast tumour tissue staining: control without lambda-phosphatase **(e)** and with lambda-phosphatase **(f)**. **Figure S4** showing Kaplan–Meier curves and multivariate Cox regression of breast cancer survival (BCS) and distant recurrence-free survival (DRFS) in the van de Vijver patient cohort, in relation to: S6K1 mRNA **(a)**; S6K2 mRNA; **(b)** S6K2 mRNA median **(c)**; and 4EBP1 mRNA **(d)**. The Cox analysis included the following variables: adjuvant chemotherapy treatment, endocrine treatment, lymph node status, and ER status. **Figure S5** showing Kaplan–Meier curves and multivariate Cox regression of breast cancer survival (BCS) in the Karolinska patient cohort, in relation to: S6K1 mRNA **(a)**; S6K2 mRNA **(b)**; and 4EBP1mRNA **(c)**. The Cox analysis included the following variables: adjuvant chemotherapy treatment, endocrine treatment, lymph node status, tumour size and ER status. **Figure S6** showing Kaplan–Meier curves and multivariate Cox regression of breast cancer survival (BCS) in the Uppsala patient cohort, in relation to S6K1 mRNA **(a)**; S6K2 mRNA **(b)**; and 4EBP1mRNA **(c)**. The Cox analysis included the following variables: adjuvant chemotherapy treatment, endocrine treatment, lymph node status, tumour size, and ER status. **Figure S7** showing an overview of suggested 4EBP1 signalling pathways, based on results from this study and previous literature. Click here for file

Additional file 2 **Is Table S1 presenting an overview of the number of patients in the different cohorts and samples available for the different analyses.** TMA, tissue microarray. Click here for file

Additional file 3 Is Table S2 presenting patient characteristics of the different cohorts included in the study. Click here for file

Additional file 4 Is Table S3 presenting the distribution of 4EBP1 and p4EBP1_S65 protein expression among samples in the Stockholm 3 cohort. Click here for file

Additional file 5 **Is Table S4 presenting 4EBP1, S6K1 and S6K2 mRNA in the Stockholm 2 cohort: correlations to gene copy number, clinicopathological factors, and the PI3K/AKT/mTOR pathway.** Tam, tamoxifen; RT, radiotherapy; CMF, cyclophosphamide–metotrexate–5-fluorouracil chemotherapy. Click here for file

Additional file 6 Is Table S5 presenting 4EBP1 and p4EBP1_S65 protein in the Stockholm 3 cohort: correlations to clinicopathological factors and the AKT/mTOR pathway. Click here for file
