# Supplementary material for: The mTOR effectors 4EBP1 and S6K2 are frequently coexpressed, and associated with a poor prognosis and endocrine resistance in breast cancer: a retrospective study including patients from the randomised Stockholm tamoxifen trials
Source: Breast Cancer Res. 2013 Oct 17;15(5):R96. doi: 10.1186/bcr3557 (PMC3978839; doi:10.1186/bcr3557)
Supplement: Additional file 1: Figure S1 — Showing patient flow through the study: the randomised Stockholm tamoxifen trial, Stockholm 2 and Stockholm 3 cohorts. Tam, tamoxifen; RT, radiotherapy; CMF, cyclophosphamide–metotrexate–5-fluorouracil chemotherapy; TMA, tissue microarray; IHC, immunohistochemistry. Figure S2 showing examples of tumours graded for 4EBP1 nuclear and cytoplasmic staining: negative/weak (a); intermediate (b); and strong staining (c); and validation of 4EBP1 antibody specificity using immunoblot with MCF7 cell lysate (d). Figure S3 showing examples of tumours graded for p4EBP1_S65 nuclear and cytoplasmic staining: negative/weak (a); intermediate (b) and strong staining (c); and validation of p4EBP1_S65 antibody specificity; immunoblot using MCF7 cell lysate (d); p4EBP1 breast tumour tissue staining: control without lambda-phosphatase (e) and with lambda-phosphatase (f). Figure S4 showing Kaplan–Meier curves and multivariate Cox regression of breast cancer survival (BCS) and distant recurrence-free survival (DRFS) in the van de Vijver patient cohort, in relation to: S6K1 mRNA (a); S6K2 mRNA; (b) S6K2 mRNA median (c); and 4EBP1 mRNA (d). The Cox analysis included the following variables: adjuvant chemotherapy treatment, endocrine treatment, lymph node status, and ER status. Figure S5 showing Kaplan–Meier curves and multivariate Cox regression of breast cancer survival (BCS) in the Karolinska patient cohort, in relation to: S6K1 mRNA (a); S6K2 mRNA (b); and 4EBP1mRNA (c). The Cox analysis included the following variables: adjuvant chemotherapy treatment, endocrine treatment, lymph node status, tumour size and ER status. Figure S6 showing Kaplan–Meier curves and multivariate Cox regression of breast cancer survival (BCS) in the Uppsala patient cohort, in relation to S6K1 mRNA (a); S6K2 mRNA (b); and 4EBP1mRNA (c). The Cox analysis included the following variables: adjuvant chemotherapy treatment, endocrine treatment, lymph node status, tumour size, and ER status. Figure S7 showing an overvi [file bcr3557-S1.ppt]

## Slide 1
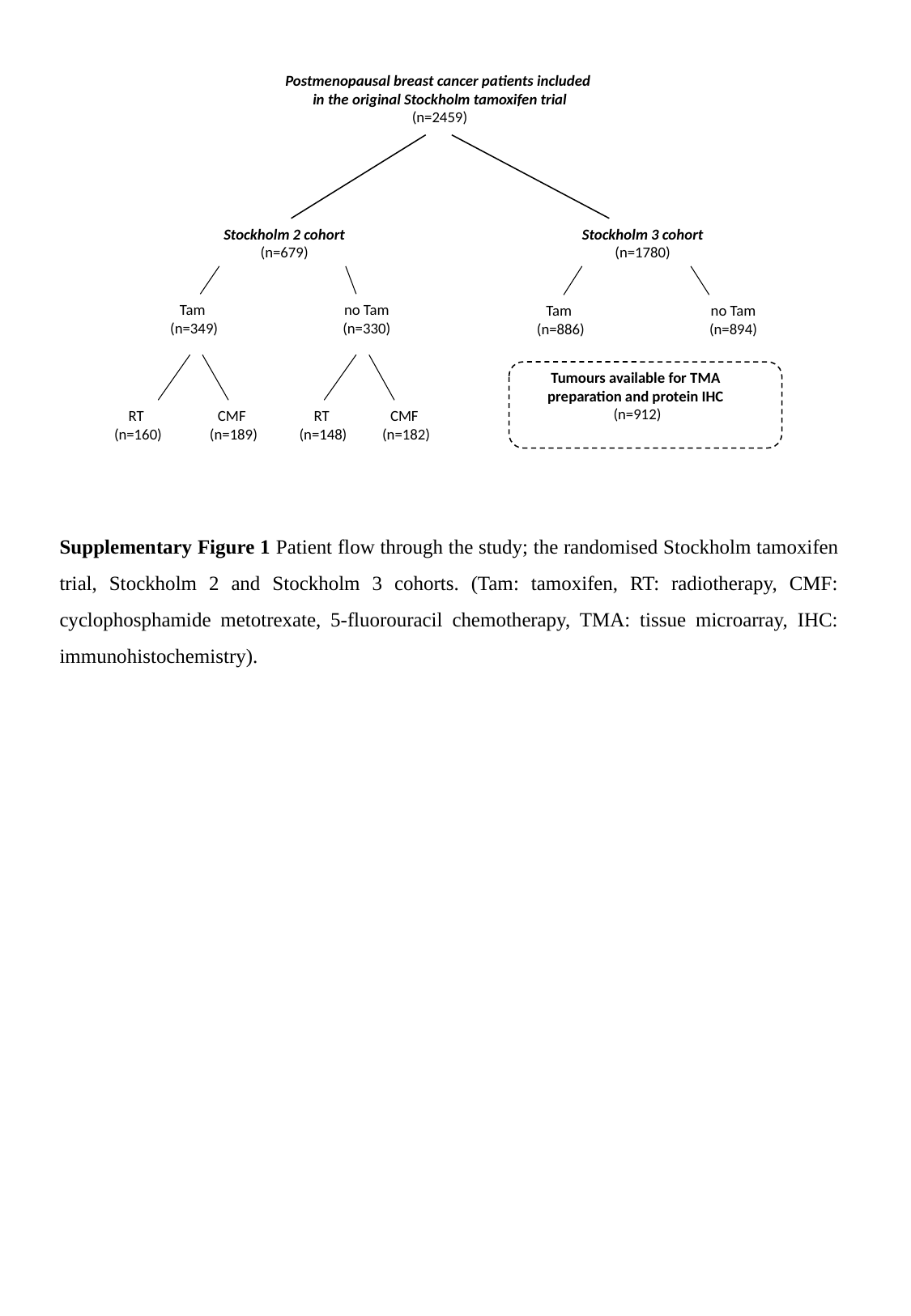

Postmenopausal breast cancer patients included
in the original Stockholm tamoxifen trial
(n=2459)
Stockholm 2 cohort
(n=679)
Stockholm 3 cohort
(n=1780)
Tam
(n=349)
no Tam
(n=330)
Tam
(n=886)
no Tam
(n=894)
Tumours available for TMA
preparation and protein IHC
(n=912)
RT
(n=160)
RT
(n=148)
CMF
(n=182)
CMF
(n=189)
Supplementary Figure 1 Patient flow through the study; the randomised Stockholm tamoxifen trial, Stockholm 2 and Stockholm 3 cohorts. (Tam: tamoxifen, RT: radiotherapy, CMF: cyclophosphamide metotrexate, 5-fluorouracil chemotherapy, TMA: tissue microarray, IHC: immunohistochemistry).

## Slide 2
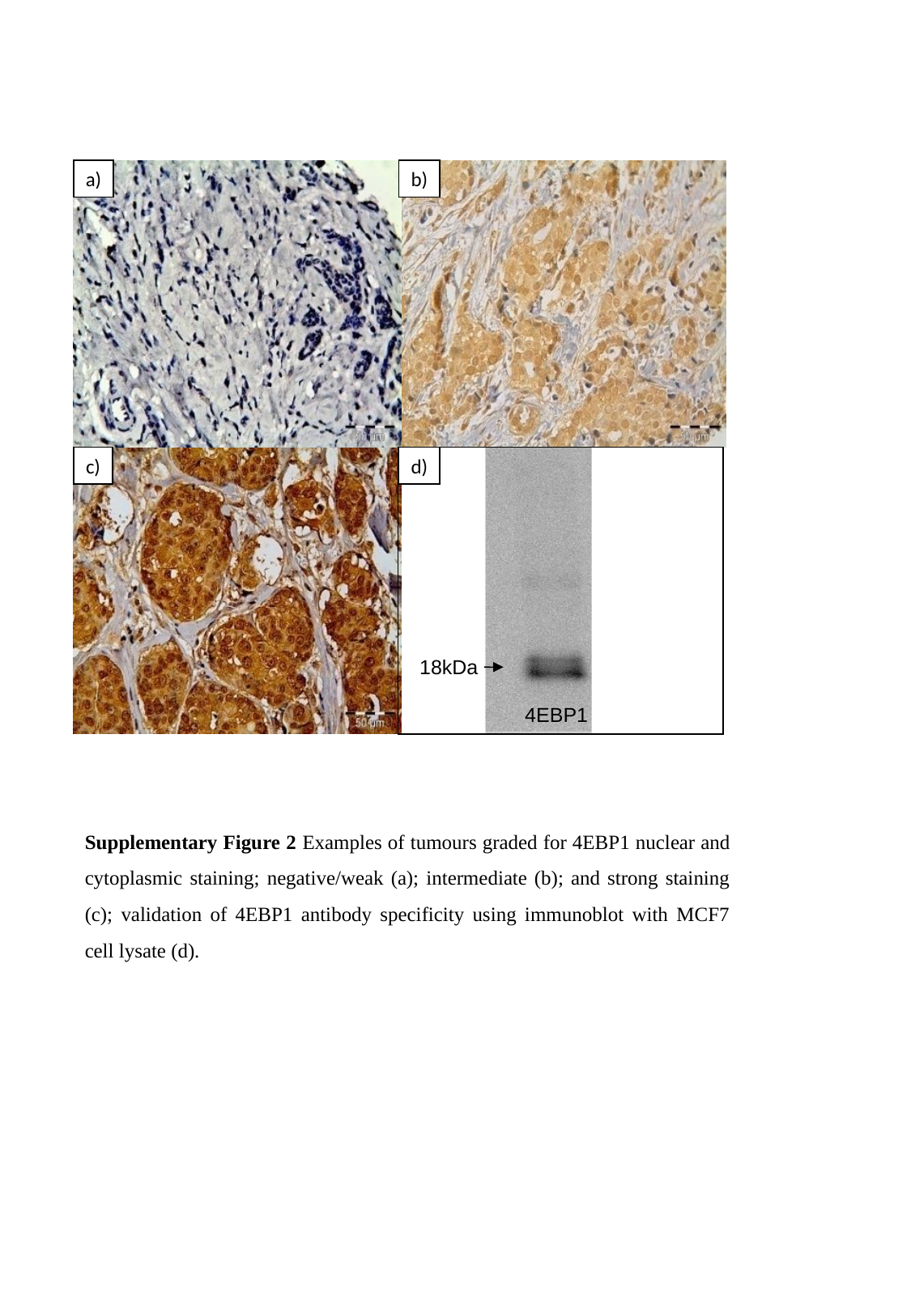

a)
b)
c)
d)
18kDa
4EBP1
Supplementary Figure 2 Examples of tumours graded for 4EBP1 nuclear and cytoplasmic staining; negative/weak (a); intermediate (b); and strong staining (c); validation of 4EBP1 antibody specificity using immunoblot with MCF7 cell lysate (d).

## Slide 3
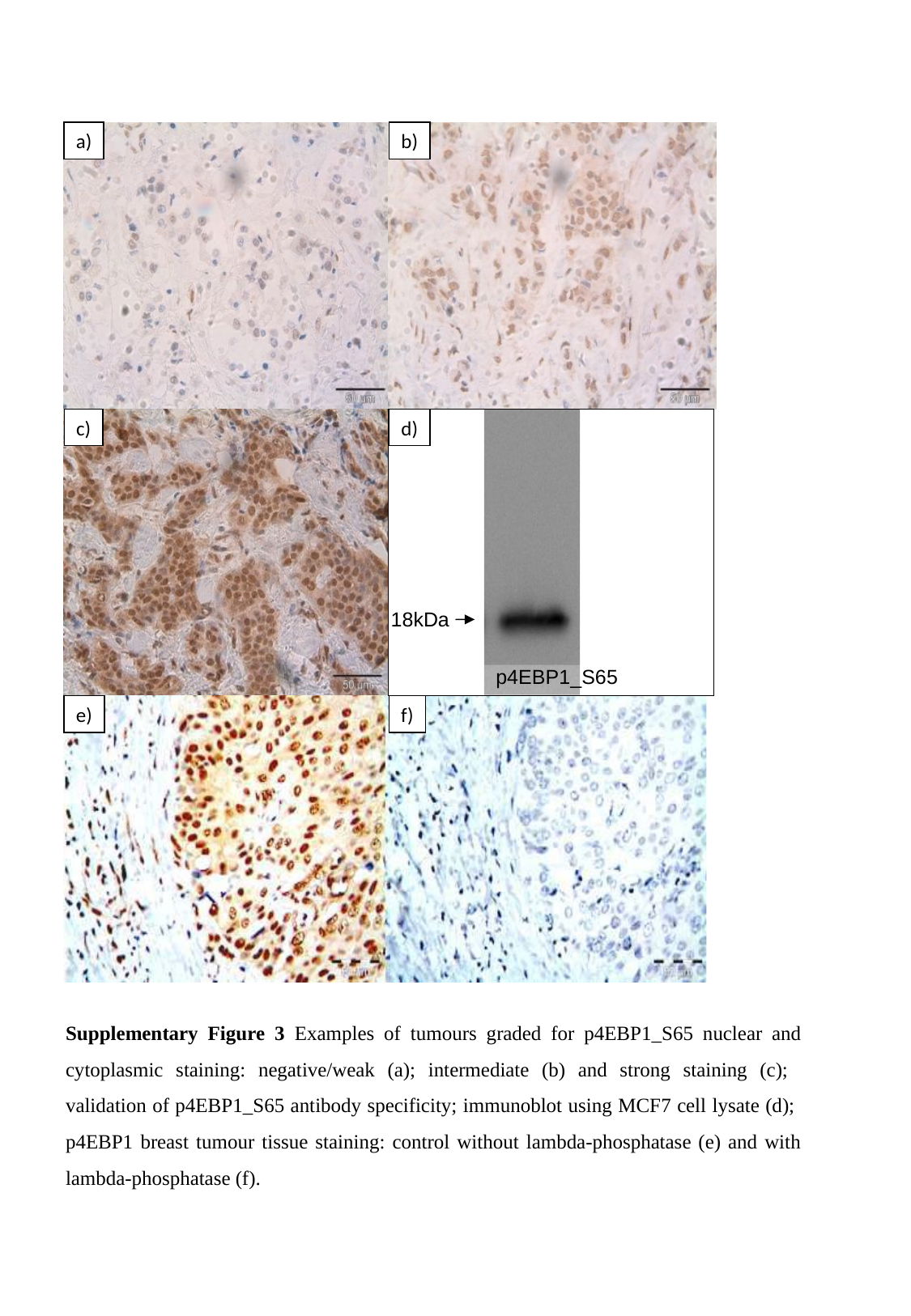

a)
b)
c)
d)
18kDa
p4EBP1_S65
e)
f)
Supplementary Figure 3 Examples of tumours graded for p4EBP1_S65 nuclear and cytoplasmic staining: negative/weak (a); intermediate (b) and strong staining (c); validation of p4EBP1_S65 antibody specificity; immunoblot using MCF7 cell lysate (d); p4EBP1 breast tumour tissue staining: control without lambda-phosphatase (e) and with lambda-phosphatase (f).

## Slide 4
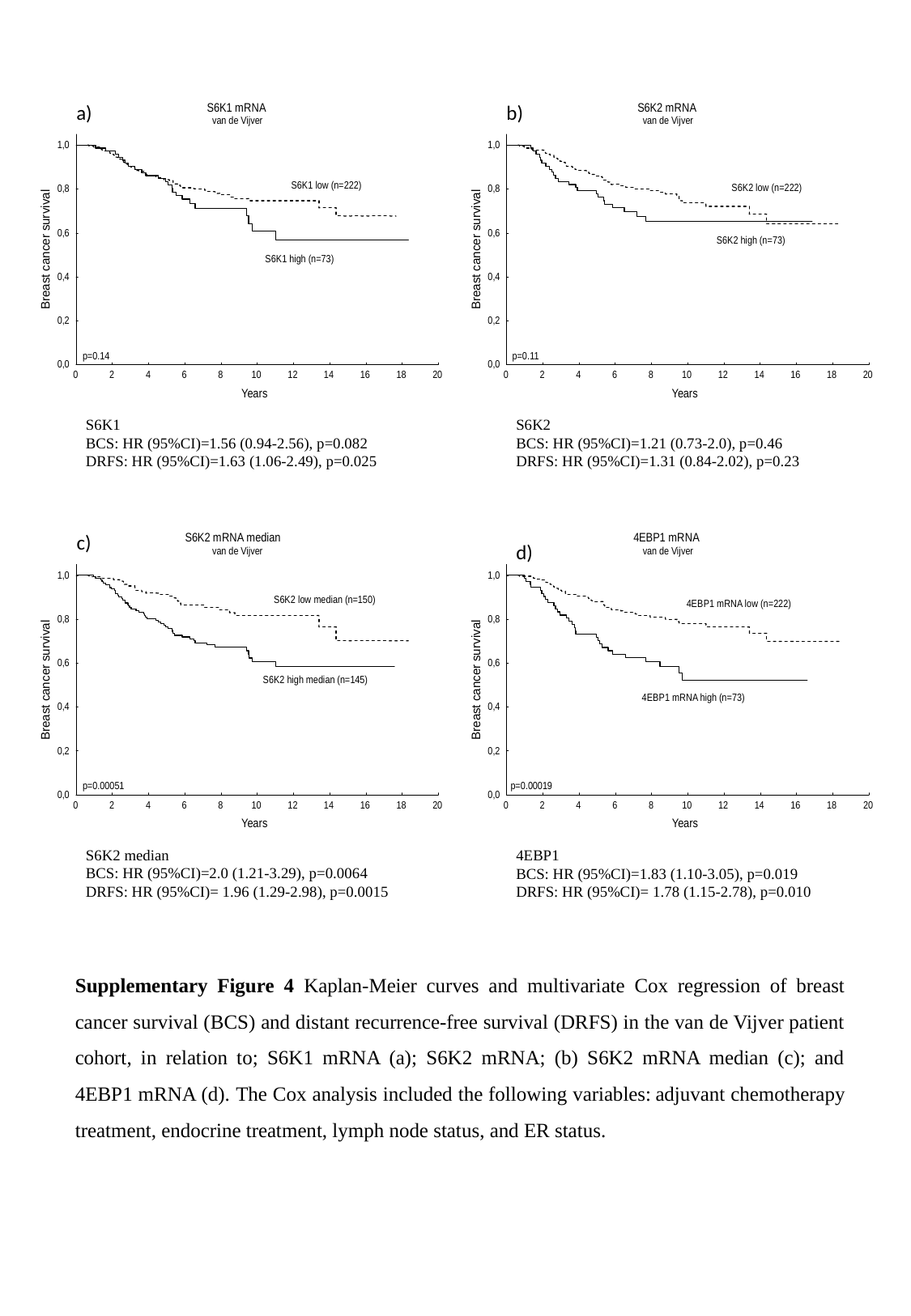

a)
b)
S6K1
BCS: HR (95%CI)=1.56 (0.94-2.56), p=0.082
DRFS: HR (95%CI)=1.63 (1.06-2.49), p=0.025
S6K2
BCS: HR (95%CI)=1.21 (0.73-2.0), p=0.46
DRFS: HR (95%CI)=1.31 (0.84-2.02), p=0.23
c)
d)
S6K2 median
BCS: HR (95%CI)=2.0 (1.21-3.29), p=0.0064
DRFS: HR (95%CI)= 1.96 (1.29-2.98), p=0.0015
4EBP1
BCS: HR (95%CI)=1.83 (1.10-3.05), p=0.019
DRFS: HR (95%CI)= 1.78 (1.15-2.78), p=0.010
Supplementary Figure 4 Kaplan-Meier curves and multivariate Cox regression of breast cancer survival (BCS) and distant recurrence-free survival (DRFS) in the van de Vijver patient cohort, in relation to; S6K1 mRNA (a); S6K2 mRNA; (b) S6K2 mRNA median (c); and 4EBP1 mRNA (d). The Cox analysis included the following variables: adjuvant chemotherapy treatment, endocrine treatment, lymph node status, and ER status.

## Slide 5
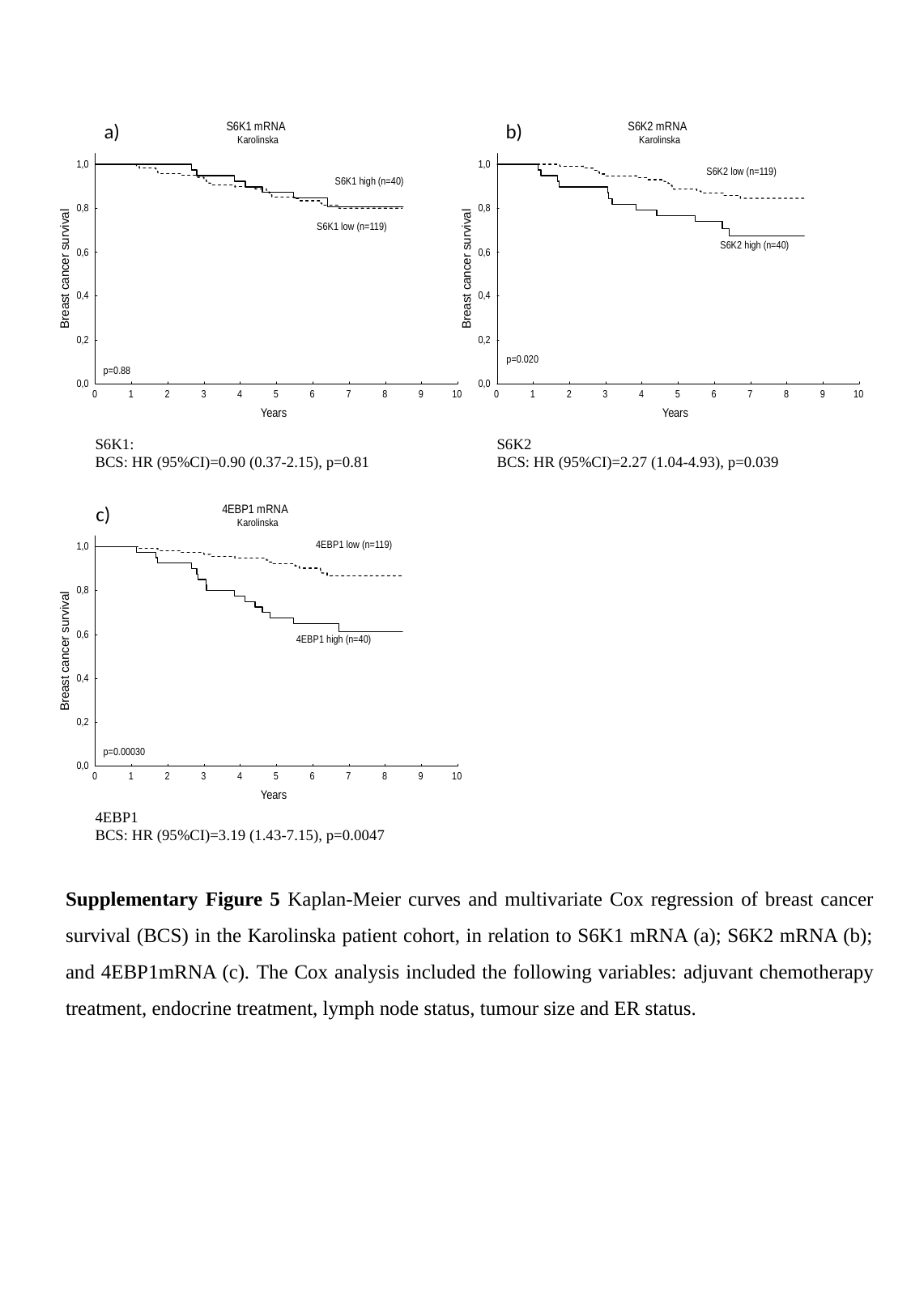

a)
b)
S6K1:
BCS: HR (95%CI)=0.90 (0.37-2.15), p=0.81
S6K2
BCS: HR (95%CI)=2.27 (1.04-4.93), p=0.039
c)
4EBP1
BCS: HR (95%CI)=3.19 (1.43-7.15), p=0.0047
Supplementary Figure 5 Kaplan-Meier curves and multivariate Cox regression of breast cancer survival (BCS) in the Karolinska patient cohort, in relation to S6K1 mRNA (a); S6K2 mRNA (b); and 4EBP1mRNA (c). The Cox analysis included the following variables: adjuvant chemotherapy treatment, endocrine treatment, lymph node status, tumour size and ER status.

## Slide 6
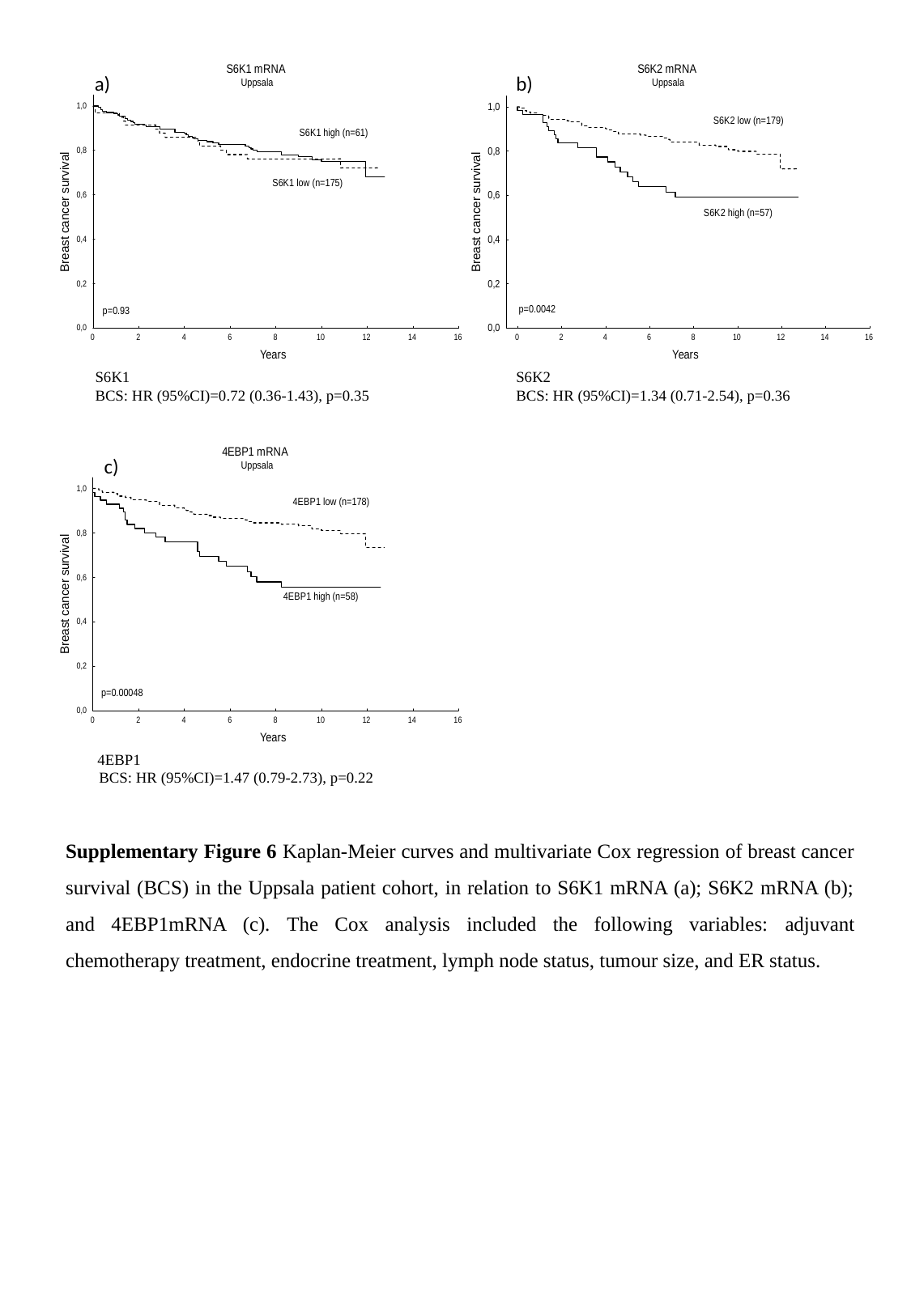

a)
b)
S6K1
BCS: HR (95%CI)=0.72 (0.36-1.43), p=0.35
S6K2
BCS: HR (95%CI)=1.34 (0.71-2.54), p=0.36
c)
 4EBP1
 BCS: HR (95%CI)=1.47 (0.79-2.73), p=0.22
Supplementary Figure 6 Kaplan-Meier curves and multivariate Cox regression of breast cancer survival (BCS) in the Uppsala patient cohort, in relation to S6K1 mRNA (a); S6K2 mRNA (b); and 4EBP1mRNA (c). The Cox analysis included the following variables: adjuvant chemotherapy treatment, endocrine treatment, lymph node status, tumour size, and ER status.

## Slide 7
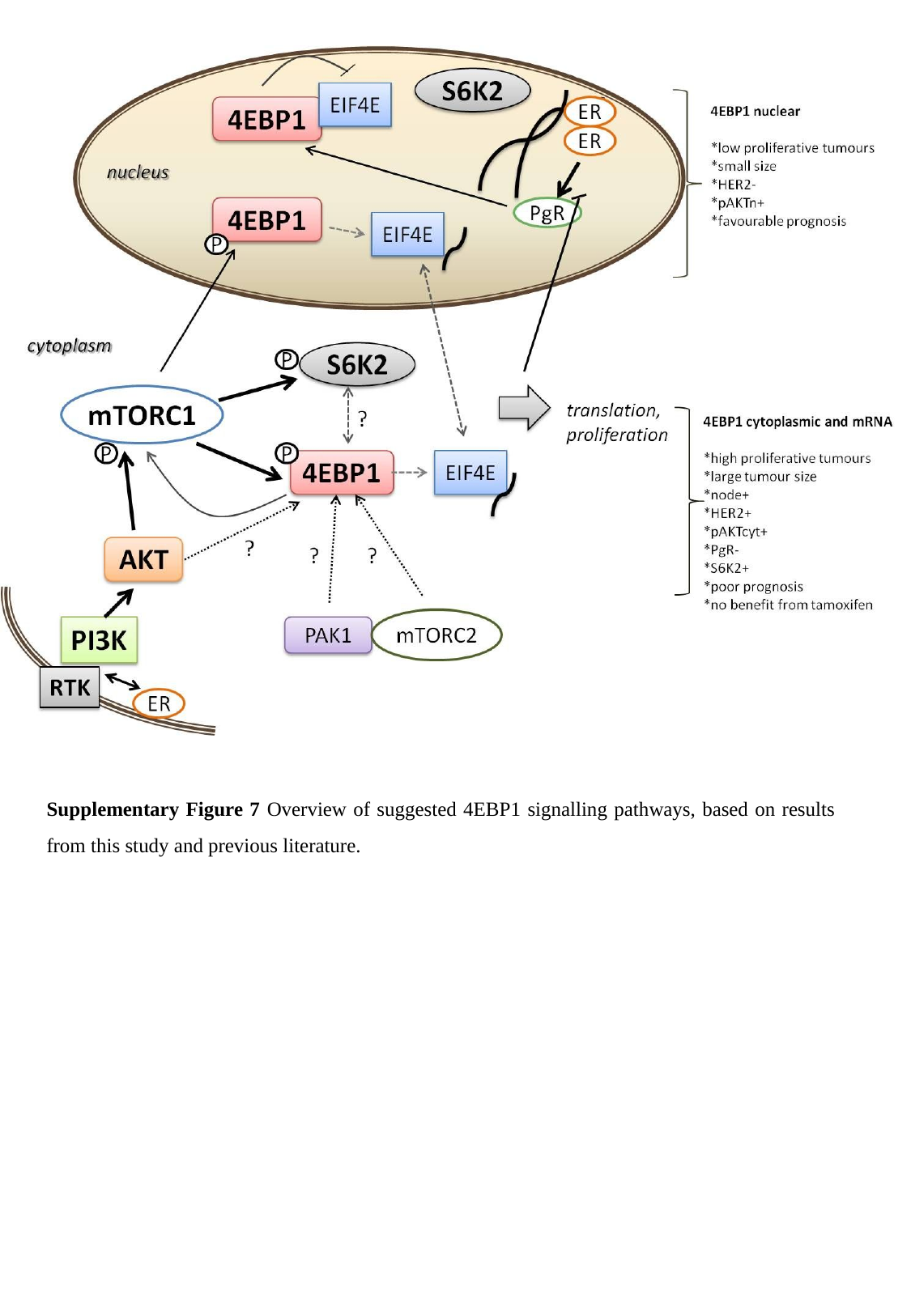

Supplementary Figure 7 Overview of suggested 4EBP1 signalling pathways, based on results from this study and previous literature.
