# Supplementary material for: The mTOR effectors 4EBP1 and S6K2 are frequently coexpressed, and associated with a poor prognosis and endocrine resistance in breast cancer: a retrospective study including patients from the randomised Stockholm tamoxifen trials
Source: Breast Cancer Res. 2013 Oct 17;15(5):R96. doi: 10.1186/bcr3557 (PMC3978839; doi:10.1186/bcr3557)
Supplement: Additional file 2 — Is Table S1 presenting an overview of the number of patients in the different cohorts and samples available for the different analyses. TMA, tissue microarray. [file bcr3557-S2.pdf]

Supplementary Table 1 Overview of no of patients in the different cohorts and samples available for the different analyses. (TMA: tissue microarray).

cohort	Stockholm 2	Stockholm 3	Van de Vijver	Uppsala	Karolinska	total
Total number of patients	679	1780	295	315	524	3593
mRNA available (n)	93	-	295	236	159	783
TMA available (n)	-	912	-	-	-	912
TMA cores available 4EBP1 (n/cyt)		739				739
TMA cores available p4EBP1_S65 (n/cyt)		768				768
