# Supplementary material for: The mTOR effectors 4EBP1 and S6K2 are frequently coexpressed, and associated with a poor prognosis and endocrine resistance in breast cancer: a retrospective study including patients from the randomised Stockholm tamoxifen trials
Source: Breast Cancer Res. 2013 Oct 17;15(5):R96. doi: 10.1186/bcr3557 (PMC3978839; doi:10.1186/bcr3557)
Supplement: Additional file 3 — Is Table S2 presenting patient characteristics of the different cohorts included in the study. [file bcr3557-S3.pdf]

**Supplementary Table 2** Patient characteristics of the different cohorts included in the study.

| | **Stockholm 2** | **Stockholm 3** | **Van de Vijver** | **Uppsala** | **Karolinska** |
|---|---|---|---|---|---|
| | **n (%)** | | | | |
| **Tumour size** | | | | | |
| **< 20 mm** | 37 (43.0) | 580 (77.2) | 155 (53.0) | 127 (25.4) | 89 (62.2) |
| **>20 mm** | 49 (57.0) | 171 (22.8) | 140 (47.0) | 124 (24.8) | 54 (37.8) |
| | | | | | |
| **Lymph node status** | | | | | |
| **-** | 6 (7.0) | 768 (100) | 151 (51.2) | 158 (65.3) | 87 (60.8) |
| + | 80 (93.0) | 0 (0) | 144 (48.8) | 84 (34.7) | 56 (39.2) |
| | | | | | |
| **Grade** | N/A | | | | |
| **1** | | 122 (18.6) | 75 (25.4) | 67 (26.9) | 27 (18.9) |
| **2** | | 382 (58.2) | 101 (34.2) | 128 (51.4) | 57 (39.9) |
| **3** | | 152 (23.2) | 119 (40.3) | 54 (21.7) | 59 (41.3) |
| | | | | | |
| **ER** | | | | | |
| **-** | 18 (20.9) | 159 (21.3) | 69 (23.4) | 34 (13.8) | 25 (17.5) |
| + | 68 (79.1) | 589 (78.7) | 226 (76.6) | 213 (88.7) | 118 (82.5) |
| | | | | | |
| **PgR** | N/A | | N/A | | N/A |
| **-** | | 327 (47.6) | | 61 (24.3) | |
| + | | 360 (52.4) | | 190 (75.7) | |
| | | | | | |
| **HER2** | | | N/A | N/A | N/A |
| **-** | 71 (77.2) | 634 (88.7) | | | |
| + | 21 (22.8) | 81 (11.3) | | | |
