# Supplementary material for: The mTOR effectors 4EBP1 and S6K2 are frequently coexpressed, and associated with a poor prognosis and endocrine resistance in breast cancer: a retrospective study including patients from the randomised Stockholm tamoxifen trials
Source: Breast Cancer Res. 2013 Oct 17;15(5):R96. doi: 10.1186/bcr3557 (PMC3978839; doi:10.1186/bcr3557)
Supplement: Additional file 4 — Is Table S3 presenting the distribution of 4EBP1 and p4EBP1_S65 protein expression among samples in the Stockholm 3 cohort. [file bcr3557-S4.pdf]

Supplementary Table 3 Distribution of 4EBP1 and p4EBP1_S65 protein expression among samples in the Stockholm 3 cohort.

4EBP1 cytoplasmic expression

	n	%
negative/weak	177	24.0
intermediate	299	40.0
strong	263	36.0
Σ	*739*	*100%*

4EBP1 nuclear expression

	n	%
negative/weak	69	9.0
intermediate	411	56.0
strong	259	35.0
Σ	*739*	*100%*

p4EBP1_S65 cytoplasmic expression

	n	%
negative/weak	203	26.4
intermediate	339	44.1
strong	226	29.4
Σ	*768*	*100%*

p4EBP1_S65 nuclear expression

	n	%
negative/weak	38	4.9
intermediate	274	35.7
strong	456	59.4
Σ	*768*	*100%*
