# Supplementary material for: The mTOR effectors 4EBP1 and S6K2 are frequently coexpressed, and associated with a poor prognosis and endocrine resistance in breast cancer: a retrospective study including patients from the randomised Stockholm tamoxifen trials
Source: Breast Cancer Res. 2013 Oct 17;15(5):R96. doi: 10.1186/bcr3557 (PMC3978839; doi:10.1186/bcr3557)
Supplement: Additional file 5 — Is Table S4 presenting 4EBP1, S6K1 and S6K2 mRNA in the Stockholm 2 cohort: correlations to gene copy number, clinicopathological factors, and the PI3K/AKT/mTOR pathway. Tam, tamoxifen; RT, radiotherapy; CMF, cyclophosphamide–metotrexate–5-fluorouracil chemotherapy. [file bcr3557-S5.pdf]

Supplementary Table 4 4EBP1, S6K1 and S6K2 mRNA in the Stockholm 2 cohort: correlations to gene copy number, clinicopathological factors, and the PI3K/AKT/mTOR pathway. Tam: tamoxifen, RT: radiotherapy, CMF: cyclophosphamide metotrexate, 5-fluorouracil chemotherapy

	**S6K1**					**S6K2**					**4EBP1**				
	**mRNA quartile n (%)**				**Spearman R**	**mRNA quartile n (%)**				**Spearman R**	**mRNA quartile n (%)**				**Spearman R**
**Gene copy number**	**0**	**1**	**2**	**3**		**0**	**1**	**2**	**3**		**0**	**1**	**2**	**3**	
0-2	18 (29.0)	14 (22.6)	15 (24.2)	15 (24.2)		17 (28.8)	19 (32.2)	12 (20.3)	11 (18.6)		N/A				
3	0 (0)	0(0)	3 (50)	3 (50)		1 (10.0)	1 (10.0)	4 (40.0)	4 (40.0)						
≥ 4	0 (0)	2 (22.2)	3 (33.3)	4 (44.4)	**R=0.30, p=0.0074**	0 (0)	0 (0)	1 (14.3)	6 (85.7)	**R=0.43, p=0.00010**					
**Tam treatment**															
no Tam	9 (23.1)	12 (30.8)	9 (23.1)	9 (23.1)		11 (28.2)	9 (23.1)	8 (20.5)	11 (28.2)		12 (30.8)	9 (23.1)	10 (25.6)	8 (20.5)	
Tam	15 (27.8)	11 (20.4)	14 (25.9)	14 (25.9)	R=0.016, p=0.88	13 (24.1)	14 (25.9)	14 (25.9)	13 (24.1)	R=0.0059, p=0.96	12 (22.2)	14 (25.9)	12 (22.2)	16 (29.6)	R=0.10, p=0.33
**CMF/RT**															
RT	9 (20.5)	10 (22.7)	10 (22.7)	15 (34.1)		9 (20.5)	12 (27.3)	10 (22.7)	13 (29.6)		13 (29.6)	8 (18.2)	8 (18.2)	15 (34.1)	
CMF	15 (30.6)	13 (26.5)	13 (26.5)	8 (16.3)	R=-0.19, p=0.074	15 (30.6)	11 (22.5)	12 (24.5)	11 (22.5)	R=-0.10, p=0.34	11 (22.5)	15 (30.6)	14 (28.6)	9 (18.4)	R=-0.06, p=0.56
**Tumour size**															
≤ 20 mm	10 (25.6)	11 (28.2)	10 (25.6)	8 (20.5)		7 (18.0)	11 (28.2)	10 (25.6)	11 (28.2)		10 (25.6)	4 (10.3)	12 (30.8)	13 (33.3)	
< 20 mm	14 (25.9)	12 (22.2)	13 (24.1)	15 (27.8)	R=0.06, p=0.60	17 (31.5)	12 (22.2)	12 (22.2)	13 (24.1)	R=-0.11, p=0.29	14 (25.9)	19 (35.2)	10 (18.5)	11 (20.4)	R= -0.17, p=0.11
**Nodes**															
-	2 (25.0)	0 (0)	1 (12.5)	5 (62.5)		1 (12.5)	2 (25.0)	1 (12.5)	4 (50.0)		1 (12.50)	3 (37.5)	2 (25.0)	2 (25.0)	
+	22 (25.9)	23 (27.1)	22 (25.9)	18 (21.2)	R=-0.17, p=0.095	23 (27.1)	21 (24.7)	21 (24.7)	20 (23.5)	R=-0.14, p=0.19	23 (27.1)	20 (23.5)	20 (23.5)	22 (25.9)	R=-0.036, p=0.73
**S-phase fraction**															
< 10%	7 (15.2)	10 (21.7)	14 (30.4)	15 (32.6)		12 (26.1)	13 (28.3)	11 (23.9)	10 (21.7)		11 (23.9)	13 (28.3)	11 (23.9)	11 (23.9)	
≥ 10%	14 (35.0)	12 (30.0)	8 (20.0)	6 (15.0)	**R=-0.29, p=0.0059**	10 (25.0)	9 (22.5)	10 (25.0)	11 (27.5)	R=0.06, P=0.58	10 (25.0)	10 (25.0)	9 (22.5)	11 (27.5)	R=0.020, P=0.85
**ER**															
-	13 (61.9)	3 (14.3)	4 (19.1)	1 (4.8)		5 (23.8)	8 (38.1)	5 (23.8)	3 (14.3)		5 (23.8)	7 (33.3)	2 (9.5)	7 (33.3)	
+	11 (15.3)	20 (27.8)	19 (26.4)	22 (30.6)	**R=0.39, p=0.00009**	19 (26.4)	15 (20.8)	17 (23.6)	21 (29.2)	R=0.098, P=0.35	19 (26.4)	16 (22.2)	20 (27.8)	17 (23.6)	R=-0.015, p=0.89
***HER2* amplification**															
-	16 (22.5)	15 (21.1)	18 (25.4)	22 (31.0)		19 (26.8)	16 (22.5)	16 (22.5)	20 (28.2)		17 (23.9)	16 (22.5)	19 (26.8)	19 (26.8)	
+	5 (29.4)	6 (35.3)	5 (29.4)	1 (5.9)	R=-0.19, p=0.076	4 (23.5)	6 (35.3)	4 (23.5)	3 (17.7)	R=-0.057, p=0.60	6 (35.3)	5 (29.4)	2 (11.8)	4 (23.5)	R=-0.11, p=0.29
**HER2 protein**															
-	15 (21.1)	14 (19.7)	20 (28.2)	22 (31.0)		18 (25.4)	17 (23.9)	17 (23.9)	19 (26.8)		19 (26.8)	18 (25.4)	17 (23.9)	17 (23.9)	
+	8 (38.1)	9 (42.9)	3 (14.3)	1 (4.8)	**R=-0.31, p=0.0024**	6 (28.6)	5 (23.8)	5 (23.8)	5 (23.8)	R=-0.034, p=0.75	5 (23.8)	5 (23.8)	5 (23.8)	6 (28.6)	R=0.045, p=0.67
***PIK3CA* mut**															
-	21 (29.2)	16 (22.2)	18 (25.0)	17 (23.6)		17 (23.6)	15 (20.8)	20 (27.8)	20 (27.8)		16 (22.2)	20 (27.8)	17 (23.6)	19 (26.4)	
+	2 (10.5)	7 (36.8)	4 (21.1)	6 (31.6)	R=0.11, p=0.29	7 (36.8)	7 (36.8)	1 (5.3)	4 (21.1)	R=-0.17, p=0.10	8 (42.1)	2 (10.5)	4 (21.1)	5 (26.3)	R=-0.082, p=0.44
**pAKT_S473**															
0%	9 (22.0)	14 (34.1)	9 (22.0)	9 (22.0)		13 (31.7)	4 (9.8)	11 (26.8)	13 (31.7)		13 (31.7)	9 (22.0)	8 (19.5)	11 (26.8)	
1-10%	5 (25.0)	4 (20.0)	7 (35.0)	4 (20.0)		8 (40.0)	5 (25.0)	3 (15.0)	4 (20.0)		4 (20.0)	8 (40.0)	5 (25.0)	3 (15.0)	
> 10%	10 (31.3)	5 (15.6)	7 (21.9)	10 (31.3)	R=0.036, p=0.73	3 (9.4)	14 (43.8)	8 (25.0)	7 (21.9)	R=-0.0090, p=0.93	7 (21.9)	6 (18.8)	9 (28.1)	10 (31.3)	R=0.099, p=0.34
