# Supplementary material for: The mTOR effectors 4EBP1 and S6K2 are frequently coexpressed, and associated with a poor prognosis and endocrine resistance in breast cancer: a retrospective study including patients from the randomised Stockholm tamoxifen trials
Source: Breast Cancer Res. 2013 Oct 17;15(5):R96. doi: 10.1186/bcr3557 (PMC3978839; doi:10.1186/bcr3557)
Supplement: Additional file 6 — Is Table S5 presenting 4EBP1 and p4EBP1_S65 protein in the Stockholm 3 cohort: correlations to clinicopathological factors and the AKT/mTOR pathway. [file bcr3557-S6.pdf]

Supplementary Table 5 4EBP1 and p4EBP1_S65 protein in the Stockholm 3 cohort: correlations to clinicopathological factors and the AKT/mTOR pathway.

**4EBP1 cytoplasm**	**Spearman R**	**p-value**
Tamoxifen treatment	-0.01	0.82
Tumour size > 20mm	0.11	0.0037
Nottingham grade	0.22	<0.0000001
ER	-0.13	0.0006
PgR	-0.07	0.082
HER2	0.13	0.00096
pAKTcyt	0.20	<0.0000001
pmTORcyt	0.02	0.61
S6K2cyt	0.20	<0.0000001
p4EBP1_S65 cyt	0.39	<0.0000001

**4EBP1 nucleus**	**Spearman R**	**p-value**
Tamoxifen treatment	0.00	0.98
Tumour size > 20mm	0.04	0.29
Nottingham grade	0.07	0.09
ER	-0.03	0.48
PgR	0.01	0.75
HER2	0.04	0.33
pAKTn	0.01	0.74
pmTORn	N/A	
S6K2n	0.05	0.21
p4EBP1_s65 n	0.14	0.00028

**p4EBP1_S65 cytoplasm**	**Spearman R**	**p-value**
Tamoxifen treatment	-0.04	0.29
Tumour size > 20mm	0.09	0.015
Nottingham grade	0.34	<0.0000001
ER	-0.16	0.0000009
PgR	-0.08	0.027
HER2	0.11	0.0046
pAKTcyt	0.34	<0.0000001
pmTORcyt	0.00	0.92
S6K2cyt	0.14	0.0001
4EBP1cyt	0.39	<0.0000001

**p4EBP1_S65 nucleus**	**Spearman R**	**p-value**
Tamoxifen treatment	-0.05	0.15
Tumour size > 20mm	-0.11	0.0033
Nottingham grade	-0.08	0.044
ER	0.03	0.4
PgR	0.04	0.33
HER2	-0.09	0.022
pAKTn	0.22	<0.0000001
pmTORn	N/A	
S6K2n	0.17	0.000002
4EBP1n	0.14	0.00028
